# Pelvic Abscess with Presentation as Inability to Ambulate

**DOI:** 10.51894/001c.6439

**Published:** 2017-12-19

**Authors:** Sarah A. Damer

**Affiliations:** 1 McLaren Health Care Macomb, PGY3 Emergency Medicine Resident, Mount Clemens, MI

**Keywords:** iliopsoas syndrome, psoas abscess, intra-abdominal abscess, pelvic abscess

## Abstract

Intra-abdominal abscesses are localized collections of pus confined in the peritoneal cavity by an inflammatory barrier. They are generally classified as intraperitoneal, retroperitoneal, or visceral and develop after perforation of a hollow viscus or by extension of infection or inflammation resulting from other conditions such as appendicitis or diverticulitis. Intra-abdominal abscesses are highly variable in presentation and clinicians must have a broad differential to avoid an inaccurate diagnosis. In this paper, presenting clinical symptoms as well as diagnosis and treatment methods are discussed in the context of this atypical presentation of a pelvic abscess. This retrospective case report presents a male patient in his early 60s who presented to the emergency department with atypical symptoms of a pelvic abscess. The author obtained all diagnostic information from patient interview and electronic health record. The patient’s history of end stage renal disease and diverticulitis with colostomy placement led to this atypical presentation of an intra-abdominal abscess. The patient’s abscess abutted the iliopsoas muscle that had given rise to his referred bilateral hip pain. This report presents a case of a male in his early 60s who presented to the hospital with complaint of bilateral hip pain and inability to ambulate. Providers admitted him to an internal medicine service and he was diagnosed with a recurrent pelvic abscess extending to his left iliopsoas muscle. Completed studies had failed to demonstrate any intrinsic pathology to the hips themselves. This case demonstrated an atypical presentation of a pelvic abscess, but brought up the theory that the etiology of the symptoms could be due to referred pain to the hips from the abscess. Further studies are required to investigate the percentage of pelvic abscess patients who primarily present with a component of hip pain.

## INTRODUCTION

Intra-abdominal abscesses are localized collections of pus confined in a patient’s peritoneal cavity by an inflammatory barrier. This barrier may be comprised of omentum (i.e., fat attached to the bowels), bowel adhesions, or the organ from which the abscess originated.^1^

Many intra-abdominal abscesses develop by extension of infection or inflammation resulting from conditions such as appendicitis, diverticulitis, Crohn’s Disease, pancreatitis, pelvic inflammatory disease, or any condition causing generalized peritonitis (i.e., inflammation of the membrane lining the abdominal wall).^2^ Prior abdominal surgery is another significant risk factor.

Traumatic abdominal injuries such as lacerations and hematomas of the liver, pancreas, spleen, and intestines can also lead to abscess formation. The abscesses usually contain a mixture of bacteria from the gastrointestinal tract. Most frequent isolates include *Escherichia coli* (E. coli), *Klebsiella*, and *Bacteroides fragilis.*^2^ *Neisseria gonorrhoeae* and chlamydial species are the most common organisms involved in pelvic abscesses.^2^

Intra-abdominal abscesses are highly variable in clinical presentation, although the majority of patients appear with abdominal symptoms or symptoms of sepsis. There may include persistent abdominal pain/tenderness, distention, mass, or ileus.^1^ Nausea, anorexia, and weight loss are also common. Other signs signifying possible infection include fever, fast heart rate or leukocytosis (i.e., high white blood cell count).^1^

Physicians should have a higher suspicion for this condition in patients with predisposing primary intra-abdominal disease or those with history of abdominal surgery. If the abscess is deeply seeded, however, many of these classic features may be absent. The only initial clues may be fever, persistent gastrointestinal dysfunction, or non-localizing debilitating illness.^1^ Symptoms may be masked by analgesics (i.e., pain relievers) or empiric antibiotic administration.

One important clinical manifestation that can play a role in the presentation of an intra-abdominal abscess is referred pain. Referred pain is pain perceived at a location other than the site of the painful stimulus/origin.^3^ This type of pain is the result of a network of interconnecting sensory nerves that supplies many different tissues. When there is an injury at one place in the network, this pain can be interpreted in the brain to radiate nerves and cause pain elsewhere in the related areas of the network.^3^

In patients with subphrenic (i.e., below the diaphragm) abscesses, irritation of contiguous structures may produce shoulder pain, hiccups, non-productive cough, chest pain, shortness of breath or pneumonia. With pelvic abscesses, frequent urination, diarrhea, or tenesmus (i.e., continual feeling that one has to defecate) may occur.^1^

Diagnosis of intra-abdominal abscesses includes a combination of laboratory studies and diagnostic imaging. Complete blood count, basic metabolic panel and blood cultures can help the most in diagnosis.^1^ Blood cultures indicating polymicrobial (i.e., several bacterial strains) bacteremia strongly implicate the presence of an intra-abdominal abscess.^1^ Plain abdominal radiograph are rarely diagnostic but may frequently indicate the need for further investigation if certain abnormalities are present. These may include localized ileus, extraluminal gas, air-fluid levels or displacement of organs.

Ultrasonography is a readily available, portable, inexpensive test and the findings can be quite specific when correlated with the patient’s clinical signs. In experienced hands, ultrasonography has an accuracy rate greater than 90% for diagnosing intra-abdominal abscesses.^1^ CT of the abdomen and pelvis with oral and intravenous contrast is the preferred diagnostic modality with greater than 95% accuracy.^1^

Appropriate treatment by clinicians can be frequently delayed due to the obscure nature of many conditions resulting in abscess formation, making diagnosis and localization difficult. Treatment modalities include antibiotic therapy, percutaneous drainage and surgical intervention. Pharmacologic therapy involves administration of empiric antibiotics. While colonic flora consists of near 400 species, an average of only four to six species are generally recovered from intra-abdominal infections.^4^ Combination antibiotic therapy or broad-spectrum single-agent therapy is most often recommended. Therapy can then be adjusted after report of culture results.

For patients with mild to moderate community-acquired infections and few risk factors for resistance or treatment failure, coverage for streptococci, Enterobacteriaceae, and anaerobes is sufficient. Single agent regimens include Ertapenem, Zosyn, or Timentin. Combination regimens include cefazolin, cefuroxime, ceftriaxone, cefotaxime, ciprofloxacin, or levofloxacin with Flagyl. For high-risk community infections, an agent with gram-negative activity broad enough to cover pseudomonas and Enterobacteriaceae resistant to non-pseudomonal cephalosporins should be chosen. Single agent regimens may include imipenem-cilastatin, meropenem, or Zosyn. Combination regimens include cefepime, ceftazidime, ciprofloxacin or levofloxacin with Flagyl. In health-care associated infections with known colonization of methicillin-resistant Staphalococcus aureus (MRSA), vancomycin should be added. In patients who are known to be colonized by highly-resistant organisms, to include vancomycin-resistant enterococci (VRE), agents such as linezolid and daptomycin should be used.^4^

Physicians should often consider an infectious disease consult. Percutaneous CT guided catheter drainage is the standard treatment of most intra-abdominal abscesses.^2^ Surgical drainage becomes mandatory when residual fluid cannot be evacuated with catheter irrigation, manipulation, or additional drain placement.

Intra-abdominal abscesses have a mortality rate of 10 to 40%.^5^ Outcome depends on the patient’s primary illness and general medical condition rather than on the specific nature and location of the abscess. Risk factors include multiple surgical procedures, age older than 50 years, delay in initial intervention greater than 24 hours, immunocompromised conditions, poor nutritional status, multiple organ failure, and complex, recurrent, or persistent abscesses.^4^ Multiple organ failure is a primary cause of death.^4^

### Case Report

A Caucasian male in his early 60s presented to the McLaren Macomb Emergency Department (ED) with a complaint of bilateral hip pain and inability to ambulate. The pain had been present for the past four-to-five days and was progressive in nature. The patient admitted to being fairly active and normally able to ambulate without the assistance of a cane or walker up until symptoms began. He described his pain as 10 out of 10 constant pain in the anterior bilateral hip joints with mild radiation into his lumbar back and groin, with no radiation into his legs. His pain increased with ambulation but improved with rest. He attempted to take three doses of aspirin at home without any relief in his symptoms and began using a walker to help him ambulate.

The patient had an extensive medical history. This history included sigmoid diverticulitis with sigmoid colectomy in 2012, deep vein thrombosis, duodenal ulcer, benign prostatic hypertrophy and hypertension. He also suffered from chronic obstructive pulmonary disease, chronic thrombocytopenia, peripheral vascular disease, atherosclerotic coronary artery disease and paroxysmal atrial fibrillation. He had been diagnosed with end stage renal disease and was on hemodialysis. He subsequently had multiple hemodialysis catheter infections with bacteremia (MRSA), obstructive uropathy with chronic hydronephrosis, and several peritoneal and pelvic abdominal abscesses. He denied having noticed any recent changes in his ostomy output.

The patient’s past surgical history included inferior vena cava filter placement, cholecystectomy, sigmoid colectomy with end colostomy, numerous cystoscopies, bilateral ureteral stents, peroneal abscess incision and drainage, esophagogastroduodenoscopy, colonoscopy, several IR drainages of pelvic abscesses, exploratory laparotomy with enterolysis, temporary catheter placement, and numerous arteriovenous fistula creations.

The patient took amiodarone, Prilosec, Imdur, and aspirin. He had no allergies to any medications. He denied use of nicotine, alcohol or any illicit drugs. He lived with and took care of his elderly mother. His father had died from a cerebral vascular accident and his mother had a history of coronary artery disease.

Physical exam revealed a well-nourished, well-hydrated male who was in no acute distress. He had been brought from home via ambulance as ambulation was too painful. His vital signs demonstrated hypotension with a blood pressure of 71/51. Patient admits that this was his typical baseline. His pain was worse on the right than the left. He had increased tenderness to log roll, axial compression, and passive flexion of the right hip when compared to the left.

He had intact two-point discrimination and light touch in the L2-S1 nerve distributions bilaterally. He had intact +2 out of 4 dorsalis pedis and posterior tibial pulses found with Doppler ultrasound. He elicited 5 out of 5 muscle strength in his bilateral lower extremities. There was no tenderness to palpation over the greater trochanteric region of either hip. He had a negative straight leg raise bilaterally. The patient had severe brawny edema and dryness to his lower extremities.

**Image 1:**
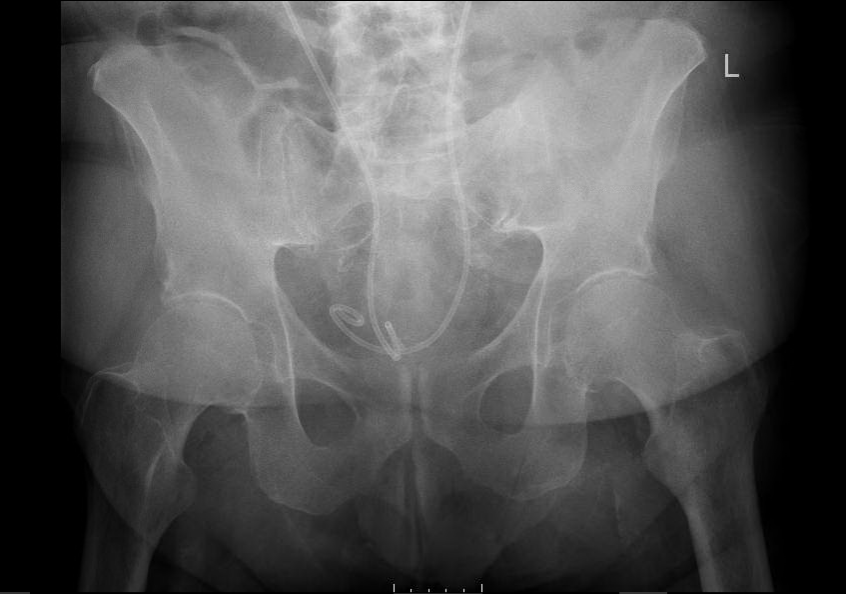
Pelvis X-ray

Laboratory studies demonstrated a normocytic anemia, chronic thrombocytopenia, hyponatremia, and chronic kidney disease. His sedimentation rate and c-reactive protein levels were elevated. When the patient was sent over for x-ray imaging, left hip, right hip, and pelvis x-rays demonstrated no abnormalities. Lumbosacral x-ray demonstrated mild scoliosis with diffuse moderate degenerative change. (Image 1) The patient was offered pain medication but refused any other treatment. He was admitted to the hospital due to his intractable bilateral hip pain and inability to ambulate. Orthopedics, Nephrology, and Physical Therapy/Occupational Therapy consults were placed.

**Image 2:**
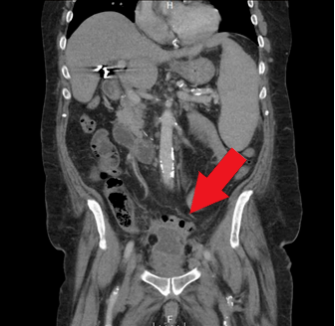
Fluid Collection - 4.9 x 3.8 cm.

**Image 3:**
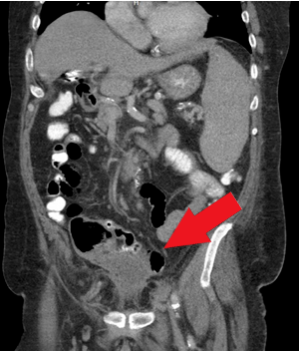
Pelvic Abscess without Signs of Communication with Bowel.

Orthopedics was the first service to evaluate the patient on hospital Day 2 and ordered a CT of the abdomen and pelvis with contrast. This study found that superior to the bladder, there was a 4.9 x 3.8 cm fluid collection with peripheral enhancement concerning for abscess. (Image 2) Internal Medicine then evaluated the patient and reviewed the results of the CT scan. Blood cultures were obtained and the General Surgery service was consulted. A repeat CT with oral contrast was ordered by General Surgery on hospital Day 3. This test showed that the adjacent loops of the intestines along the anterior and superior margin of the fluid collection were partially opacified without obvious extravasation of oral contrast in the region, suggesting fistula (i.e., communication with the bowel). (Image 3)

The blood cultures grew out E. coli x 2 after 24 hours. The patient was placed on Zosyn and vancomycin. On hospital Day 5, Interventional Radiology performed CT guidance drainage of the pelvic abscess with catheter placement. Culture results would later grow E. coli. The Urology service was also consulted as the patient had history of hydronephrosis due to obstructive uropathy with bilateral stent placement. This service was asked to evaluate the need for stent replacement and integrity of the bladder with its close approximation to the abscess.

On hospital Day 6, the patient went to the operating room with Urology for a bilateral retrograde urogram. The study demonstrated findings consistent with communication of the urinary bladder with the abscess cavity previously drained. The stents were exchanged, a urinary catheter was placed and a urine sample was obtained for which culture results came back positive for E.coli. The patient remained on Zosyn.

As the patient continued to experience significant bilateral hip pain, an MRI of the patient’s thoracic/lumbar spine and bilateral hips was ordered on Day 7. A thoracic spine MRI demonstrated a small central disc protrusion at the Thoracic 4-T5 level. No spinal canal stenosis or foraminal encroachment. The MRI of the lumbar spine and bilateral hips was unable to be completed since the patient could not tolerate the pain long enough to have the study performed.

The pelvic drain was removed between hospital Days 9 and 11, although progress notes are unclear as to the specific day. On hospital Day 15, the patient was discharged to a subacute rehabilitation unit on a two-week course of oral Augmentin. He had worked with Physical Therapy throughout his hospital stay. The patient slowly regained some ability to ambulate but it is unknown whether or not this was from resolving his pelvic abscess or due to rehabilitation with Physical Therapy. He was to follow up with the urologist in two-to-three weeks for urinary catheter removal.

## DISCUSSION

This case describes a patient who suffered from the inability to ambulate who was eventually diagnosed with an intra-abdominal abscess. This patient had presented to the emergency department with bilateral hip pain which would be an unusual presenting symptom for his diagnosed pathology. As earlier described, most common complaints associated with an intra-abdominal abscess include abdominal pain/tenderness, distention, nausea, anorexia, fever, fast heart rate or high white blood cell count. The patient met none of these criteria. Throughout the patient’s hospital stay, he never had a complaint related to his gastrointestinal system. On Day 3 of his hospitalization, he spiked a white blood cell count but remained afebrile throughout his course. The question arises as to whether the patient’s complaint has any relation to the intra-abdominal abscess found on CT imaging.

After an in-depth review of the patient’s medical record, it was revealed that he had an extensive medical history. The patient has suffered from diverticulitis with perforation and abscess formation. He had several drainages of the abscess completed by an interventional radiologist prior to, and after his sigmoid colectomy with end ileostomy that was performed in September 2012.

Throughout his multiple hospitalizations, he had recurrence of an intraperitoneal abscess that was located superior to the bladder and adjacent to the left iliopsoas muscle. Several studies showed altered attenuation of the iliopsoas muscle and evidence that the abscess was contiguous with the muscle. CT imaging conducted in December, 2012 indicated that the abscess involved the left iliopsoas bursa. It is this involvement/extension for which the patient’s presenting complaint could possibly have relation to his found pathology, however no direct data concerning this existed.

The psoas muscle arises from the transverse processes and lateral aspects of the vertebral bodies between the 12^th^ thoracic and 5^th^ lumbar vertebrae. It courses downward passing deep to the inguinal ligament and anterior to the hip joint capsule to form a tendon that inserts into the lesser trochanter of the femur. The iliacus muscle joins the psoas to insert via the same tendon. These two muscles are the main hip flexors. The tendon is separated from the hip capsule by the iliopsoas bursa. This bursa is the largest bursa in the body, and exists to help reduce rubbing between the iliopsoas muscle and the femur. This bursa communicates with the hip joint space in up to 15% of persons which may facilitate spread of infection between these sites.^6^

The iliopsoas muscle can be involved in several pathologic conditions to include iliopsoas tendinitis/bursitis, iliopsoas syndrome and psoas abscesses. Although uncommon injuries, iliopsoas tendinitis/bursitis occurs when the tendon and bursa becomes inflamed. They are overuse injuries that result from overloading the hips with repetitive movements.^7^ People who participate in activities such as golf, hockey, cheerleading, gymnastics, and resistance training are most susceptible to injury. Iliopsoas syndrome frequently begins as a bilateral muscle spasm which eventually becomes prominent on one side.

Psoas abscesses are a collection of pus in the iliopsoas muscle compartment which may arise from contiguous spread from adjacent structures or by hematogenous route from a distant site.^6^ They are divided into primary and secondary abscesses. Primary abscess occurs as a result of hematogenous or lymphatic seeding. Risk factors include diabetes, IV drug abuse, HIV, renal failure or other forms of immunosuppression. Secondary abscesses occur as a result of a direct spread of infection to the psoas muscle from adjacent structures. The structures include vertebrae, hip arthroplasty, GI tract, aorta, and genitourinary tract.

Symptoms of iliopsoas tendinitis/bursitis and psoas syndromes include pain, tenderness, swelling, heat or redness and loss of normal mobility. Signs of psoas abscess include back or flank pain, fever, inguinal mass, limp, anorexia, and weight loss. Pain can be present in up to 91% of cases with localization to the back, flank, or lower abdomen and possible radiation to the hip and/or posterior thigh.^6^

Originally described in 1881, the classic clinical presentation of a psoas abscess included back pain, limp and fever.^8^ Newer case studies have demonstrated that these symptoms may only be present in 30% of cases.^8^ Many patients present with nonspecific complaints such as malaise, low grade fever, abdominal/flank discomfort, a flexed and externally rotated hip and pain on movement of the hip. Pain is due to irritation of muscle belly and referred pain from nerve roots L2-L4. Due to the vague and nonspecific presenting symptoms of a psoas abscess, they are commonly misdiagnosed, although data concerning the misdiagnosis rate is sparse.^8^ One case studied reported a psoas abscess that was previously misdiagnosed as a deep vein thrombosis.^9^

After review of the presenting symptoms of iliopsoas bursitis/tendonitis, syndrome and psoas abscess, the clinical conclusion can be made that this patient may have been suffering from referred pain to the hip from his pelvic abscess that abutted the left iliopsoas muscle. This is a speculation since there are no direct reports available concerning a direct correlation between this man’s pelvic abscesses and hip pain.

However, several discrepancies arose during his workup. The patient had suffered from right hip pain yet the patient’s abscess directly invaded the left iliopsoas muscle. He also had more pain when asked to perform hip flexion and preferred to lay flat on his back without axial loading. Research has found a correlation with patients suffering from psoas abscesses and that they prefer to lay with their hips flexed.^6^ In addition, the patient had presented several times in the past for recurrent pelvic abscess and failed to complain of hip pain during those admissions. Finally, the patient may have had pathology of his lumbar spine or hips that could have been identified if the patient had been able to tolerate the MRI imaging procedure.

In support of the theory of the patient’s suffering from referred pain, the patient did experience pain upon movement of the hip and had improvement in his symptoms after IR drainage and antibiotic administration. Hip pain, especially in Crohn’s patients should prompt consideration of a psoas abscess as the incidence has been estimated to be between 0.4 and 4.3%.^6^ Psoas abscess has also been described in the setting of appendicitis, colorectal cancer, ulcerative colitis and following abdominal surgery so evidence exists with other GI pathology. While a question was made during the patient’s hospital stay as to whether his symptomatology was somehow related to his pathology versus incidental, the author does not believe the theory of referred pain is far-fetched.

## CONCLUSIONS

This report presents a case of a Caucasian male in his early 60s who presented to the emergency department with complaint of bilateral hip pain and inability to ambulate. He was admitted to the hospital and found to have a recurrent pelvic abscess which extended to his left iliopsoas muscle. The patient failed to demonstrate the typical symptoms of a pelvic abscess and therefore his pathology was not revealed initially in the emergency department. It was not until the patient had been hospitalized for a day and his medical record had been thoroughly reviewed, when his diagnosis was made. Even then, his presenting complaints could not be fully explained. Additional imaging studies failed to demonstrate any intrinsic pathology to the hips themselves.

This case demonstrated an atypical presentation of a pelvic abscess, but questions remained as to whether the patient’s symptoms were due to referred pain to the hips. Conditions such as iliopsoas tendinitis/bursitis and psoas syndromes present with pain, tenderness, swelling, heat or redness and loss of normal mobility while signs of a psoas abscess include back or flank pain, fever, inguinal mass, limp, anorexia, and weight loss. These clinical findings are more consistent with what the patient in the case study had presented to providers. Further investigations are required to determine the percentage of patients with pelvic abscesses who initially present with a component of hip pain.

### Conflict of Interest

The authors declare no conflict of interest.
